# Recombinant human adenovirus type 5 administration for the treatment of malignant ascites or pleural effusion in cancer patients: a meta-analysis

**DOI:** 10.3389/fonc.2025.1592995

**Published:** 2025-09-17

**Authors:** Changsong Duan, Xue Liu

**Affiliations:** Department of Medicine, Shanghai Sunway Biotech Co., Ltd, Shanghai, China

**Keywords:** malignant ascites or pleural effusion, recombinant human adenovirus Type 5, efficacy, safety, meta-analysis

## Abstract

**Objective:**

H101 (recombinant human adenovirus type 5) has favorable efficacy and safety in cancer patients with malignant ascites (MA) or pleural effusion (MPE). However, a comprehensive evaluation has not yet been conducted. This meta-analysis aimed to comprehensively investigate the efficacy and safety of H101 in these patients.

**Methods:**

The meta-analysis was registered on PROSPERO (ID: CRD420251052407). A comprehensive study search was conducted in PubMed, Web of Science, Cochrane Library, Wan Fang, CNKI, and SinoMed until October 2024. Studies reporting on the remission and safety results in cancer patients with MA/MPE treated with H101 were screened. The overall remission rates (ORRs) of ascites or pleural effusion and adverse reactions were analyzed.

**Results:**

A total of 13 studies involving 993 patients were included. The pooled ORR was 69.9% (95%CI = 63.5%–76.4%). The pooled rates of fever, nausea or vomiting, and leukopenia were 22.5% (95%CI = 10.2%–34.9%), 14.0% (95%CI = 6.8%–21.2%), and 24.3% (95%CI = 9.6%–39.1%), respectively. Subgroup analysis revealed that the ORR was higher in studies with a single cancer type than in those with multiple cancer types (*p* = 0.012). There was no publication bias in the ORR, the rate of nausea or vomiting, or the rate of leukopenia. The publication bias in the rate of fever was corrected using the trim-and-fill method, and the adjusted rate was 5.4% (95%CI = 0.0%–22.0%). All of the included studies were of high-quality, with a low risk of bias. The sensitivity analysis revealed high robustness of the results.

**Conclusion:**

H101 is effective and safe for the treatment of MA/MPE in patients with cancer and may be a promising modality for their clinical management.

## 1 Introduction

Malignant ascites (MA) or pleural effusion (MPE) is a common complication of various advanced tumors, which is characterized by the pathological accumulation of malignant cells in ascitic or pleural fluid (1–3). MA or MPE usually presents as abdominal distension and breathing difficulties, severely affecting the quality of life and even the survival of patients (4–6). The traditional treatment methods for MA/MPE include simple puncture and drainage, hyperthermic perfusion therapy, and intraperitoneal/intrapleural perfusion therapy with chemotherapy drugs, but these treatment modalities have shown limited efficacy and cause side effects (7, 8). Therefore, it is critical to search for more strategies for the management of MA/MPE.

Oncolytic viruses (OVs) are emerging antitumor agents that kill infected cells directly without destroying normal cells and have been gradually applied in the clinical management of multiple cancers (9, 10). H101 (recombinant human adenovirus type 5) is an adenovirus with an E1B-55-kDa gene deletion and is the first OV approved on the market in China (11–13). A variety of clinical studies have revealed that H101 is effective and is well tolerated in the treatment of MA/MPE in patients with cancer (14–26). For example, a study reported that, in patients with MA, intraperitoneal H101 administration yielded an ascites response rate of 40.0% and an ascites control rate (defined as the ratio of patients who showed no progression of ascites volume) of 75.0%, with no grade III/IV adverse events (24). Another study revealed that the overall remission rates (ORRs) of ascites or pleural effusion were 60.2%–60.4% in patients with MA/MPE who underwent H101-involved treatment (25). However, there is still a lack of a comprehensive analysis exploring the clinical benefits of H101 in these patients.

Therefore, this is the first meta-analysis aiming to systematically evaluate the efficacy and safety of H101 in cancer patients with MA/MPE.

## 2 Methods

### 2.1 Search strategy and selection criteria

The study was registered on the International Prospective Register of Systematic Reviews (PROSPERO; approval no. CRD420251052407), which was accessed at https://www.crd.york.ac.uk/PROSPERO/view/CRD420251052407. The detailed protocol for this meta-analysis has not been published. A comprehensive methodological workflow diagram of this meta-analysis is shown in **Supplementary Figure S1**.

A thorough study search was conducted in PubMed, Web of Science, Cochrane Library, Wan Fang, CNKI, and SinoMed until October 2024. The search used free text terms and keywords, including “cancer, carcinoma, neoplasms, malignant, ascites, pleural effusion, human type 5 recombinant adenovirus, oncolytic viruses, and H101.” The search strategy was adapted to the unique retrieval methods of the different databases. As an example, a full search strategy for PubMed is shown in **Appendix 1**. Two researchers independently screened the studies, and studies were selected for further evaluation.

The inclusion criteria were as follows: 1) studies that reported on cancer patients with MA/MPE; 2) studies that reported on the remission and safety results of MA/MPE treated with H101; and 3) studies published in English or Chinese. The exclusion criteria were as follows: 1) reviews or meta-analyses; 2) case reports; and 3) academic dissertations. In addition, gray literature or clinical trial registries were not taken into consideration as their results have not yet been reviewed by peer experts and their quality cannot be guaranteed.

A total of 13 studies were included in this meta-analysis, and ethical approval status was reviewed for all studies. Four studies explicitly reported obtaining ethical approval (21, 24–26), while this information was unavailable for the remaining nine studies (14–20, 22, 23).

### 2.2 Data extraction and outcomes

The following data were extracted: 1) publication details, including the first author’s name and year; 2) study design details, including the study type and sample size; 3) patient details, including age, male cases, disease type, and cancer type; and 4) treatment details, including therapy type and drugs. The outcomes included the ORR and adverse reactions. The ORR was defined as the complete remission (CR) rate plus the partial remission (PR) rate of ascites or pleural effusion. CR and PR were determined according to a previous study (27). Specifically, CR was defined as accumulated effusion that had disappeared and remained stable for at least 4 weeks, while PR was defined as accumulated effusion that had decreased by 50%, was associated with improved symptoms with no increased accumulation of fluid, and remained stable for at least 4 weeks (27). Adverse reactions included fever, nausea or vomiting, and leukopenia. In this meta-analysis, for the studies that reported both the H101-related group and the control group, only the data of the H101-related group were extracted for analysis. Moreover, outcomes that were reported and available from three or more studies were used for pooling the effect size.

### 2.3 Quality assessment

The Newcastle–Ottawa Scale was used to assess non-randomized controlled trials (RCTs), which were assessed from three domains and scored for each domain. The total score was 9 points, and the higher the score, the better the quality (28). The Cochrane ROB tool was applied to evaluate the RCTs, which were assessed from six items, with each item categorized as low risk, unclear, or high risk. The Cochrane ROB tool is available at Risk of Bias 2 (RoB 2) tool|Cochrane Methods (https://methods.cochrane.org/risk-bias-2).

### 2.4 Data analysis

R software version 4.3.3 was used for data analysis. Proportions with 95% confidence intervals (CIs) were used for data synthesis. A random effects model was used when *I* ^2^ exceeded 50%, indicating the presence of heterogeneity. Subgroup analysis was used to explore the heterogeneity among studies. Specifically, predefined and exploratory subgroup analyses were conducted to investigate potential sources of heterogeneity: 1) therapy type (monotherapy *vs*. combination therapy), based on the expected impact of different treatment regimens on the variability of the results; 2) publication year (before 2015 *vs*. after 2015), based on the exploration of the impact of studies published in the past 10 years *versus* those published 10 years ago on the variability of the results; 3) study design (non-RCT *vs*. RCT), based on the expected impact of randomization on the variability of the results; and 4) cancer type (multiple *vs*. single), based on the exploration of the impact of different study subjects on the variability of the results. All subgroup analyses used random-effects models due to the expected residual heterogeneity. Multivariate meta-regression was performed to further explore the sources of heterogeneity, including the therapy type, the publication year, the study design, and the cancer type. Begg’s test was applied for evaluation of publication bias, and a trim-and-fill method was utilized if publication bias existed. A funnel plot was used to visualize the publication bias. Sensitivity analysis was performed to evaluate the robustness of the model via omitting studies one by one. A *p*-value <0.05 indicated significance.

## 3 Results

### 3.1 Study screening process

**Figure 1:**
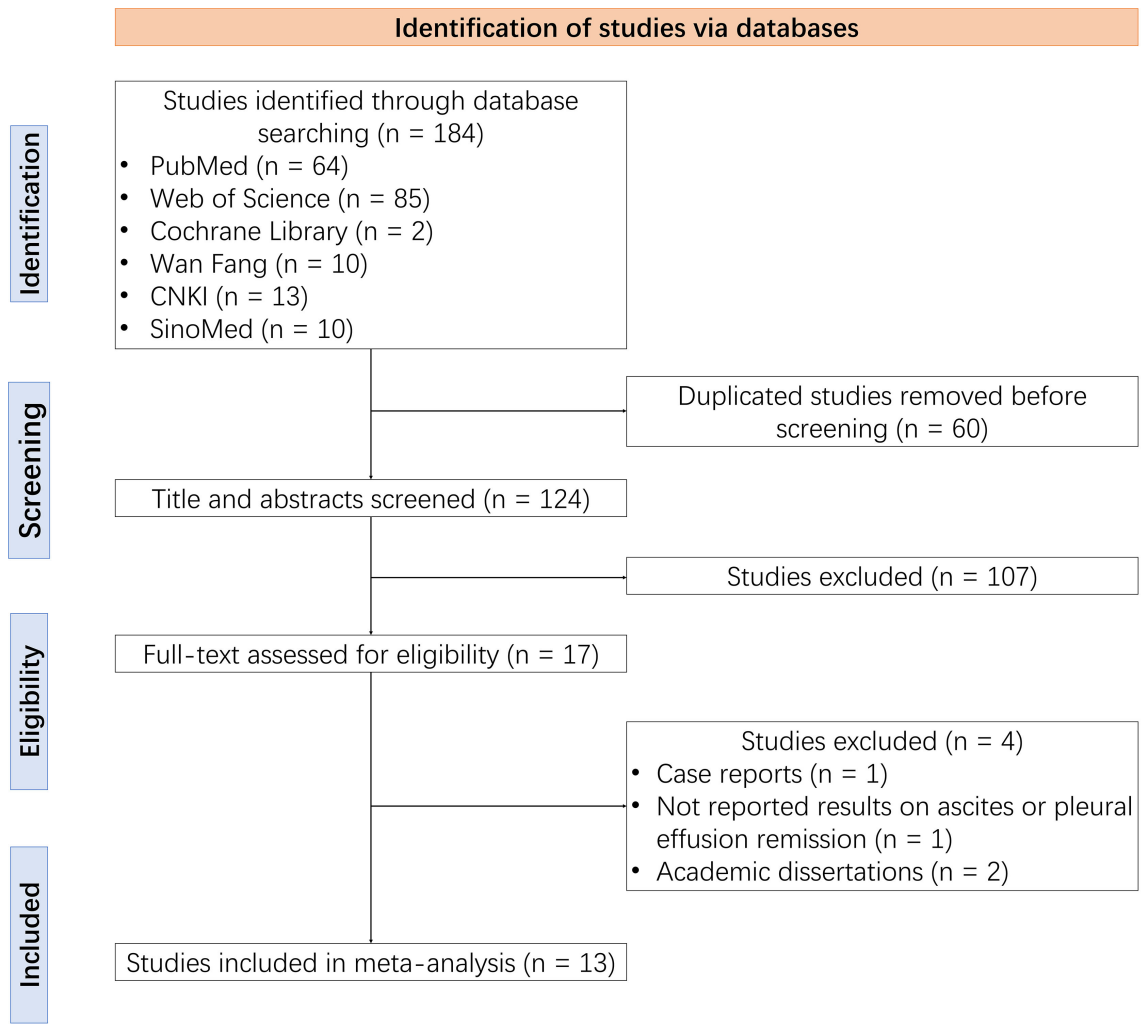
Study selection flowchart.

A total of 184 studies were identified through the database search, from which 60 duplicate studies were removed before screening. After screening the titles and abstracts, 107 studies were excluded. The remaining 17 studies were assessed for eligibility through reading the full text, with a further four ineligible studies removed. Ultimately, 13 studies were included in this meta-analysis (14–26) (**Figure 1**).

### 3.2 Information on the included studies

The 13 included studies involving 993 cases were published between 2009 and 2024. There were five observational studies and eight RCTs. In terms of cancer type, five studies included patients with multiple cancers, and eight studies included patients with a single cancer. In addition, seven studies included patients with monotherapy, four studies involved patients with combination therapy, and two studies included both. Further detailed information on the included studies is presented in **Table 1**.

**Table 1:** Characteristics of the included studies.

First author’s name	Publication year	Study type	Sample size	Age (years)	Male cases	Disease type	Cancer type	Therapy type	Drug type
HL Liu (1) (14)	2009	Observational	4	Mean: 52.0	3	MA	Multiple	Monotherapy	H101
HL Liu (2) (14)	2009	Observational	5	Mean: 57.6	1	MA	Multiple	Combination	H101+others
XJ Liu (15)	2010	RCT	23	Mean: 57.5	NR	MPE	Single	Monotherapy	H101
SL Wu (16)	2011	Observational	9	Mean: 52.2	5	MPE	Single	Combination	H101+others
X Cheng (17)	2012	RCT	25	Range: 38.0–76.0	NR	MPE	Multiple	Combination	H101+others
F Yang (18)	2013	RCT	26	Mean: 64.0	16	MPE	Single	Monotherapy	H101
L Gong (19)	2014	Observational	25	Mean: 65.7	17	MPE	Multiple	Combination	H101+others
ZX Zhan (20)	2015	RCT	52	Mean: 65.5	30	MPE	Single	Monotherapy	H101
GF Chen (21)	2017	RCT	25	Mean: 65.3	17	MPE	Single	Monotherapy	H101
X Tang (22)	2017	RCT	52	Mean: 65.6	30	MPE	Single	Monotherapy	H101
W Wang (23)	2018	RCT	30	Mean: 49.0	17	MPE	Single	Monotherapy	H101
YL Zhang (24)	2022	Observational	40	Median: 62.0	27	MA	Multiple	Monotherapy	H101
BC Wang (1) (25)	2023	Observational	467	Median: 60.0	165	MA/MPE	Multiple	Monotherapy	H101
BC Wang (2) (25)	2023	Observational	176	Median: 61.5	64	MA/MPE	Multiple	Combination	H101+others
HG Liu (26)	2024	RCT	34	>60.0 years: 16 cases	25	MA	Single	Combination	H101+others

*H101*, recombinant human adenovirus type 5; *RCT*, randomized controlled trial; *NR*, not reported; *MA*, malignant ascites; *MPE*, malignant pleural effusion.

### 3.3 Overall remission rate

**Figure 2:**
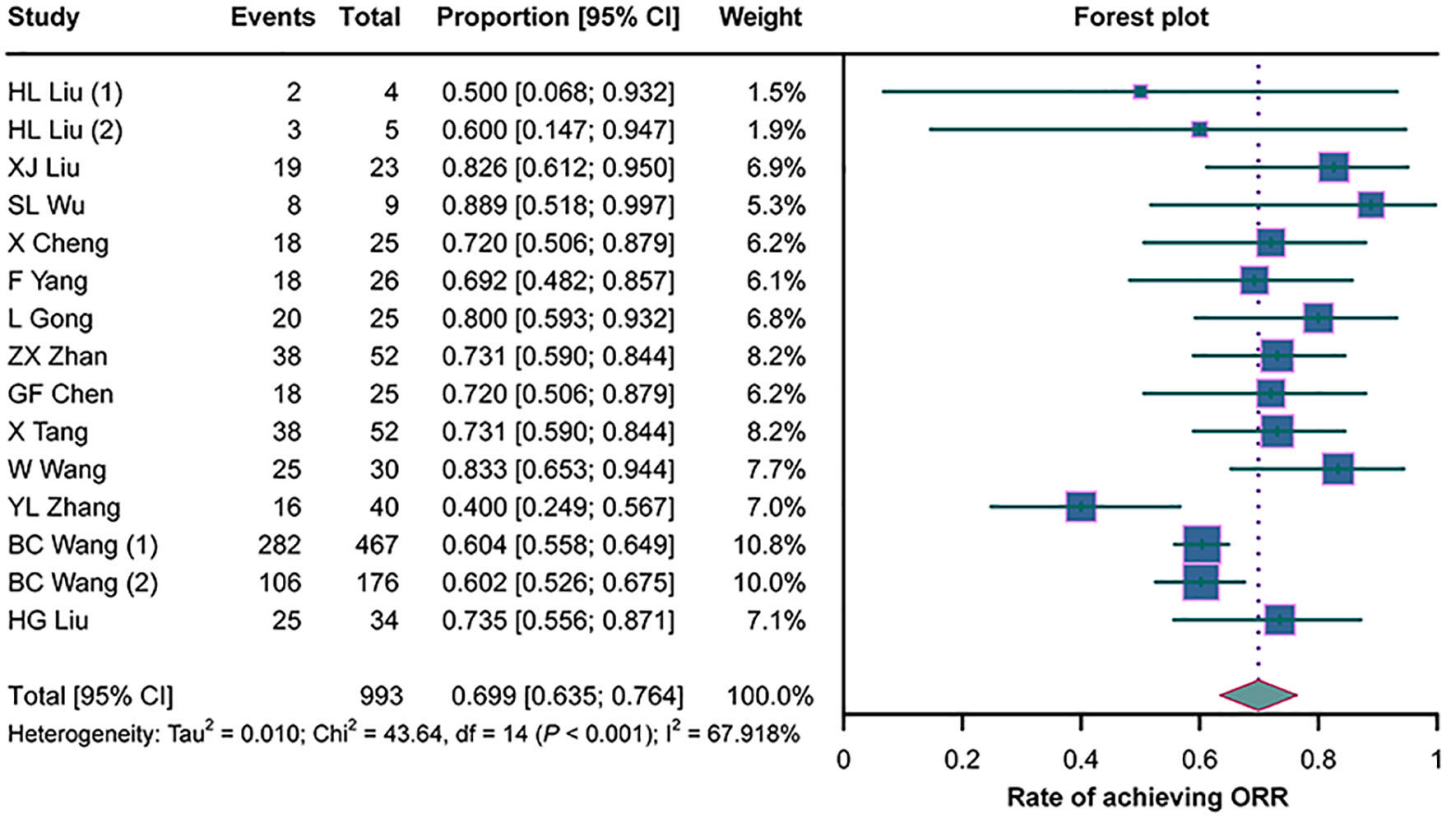
Forest plots of the overall remission rates (ORRs) in cancer patients with malignant ascites (MA) or pleural effusion (MPE) treated with H101.

All 13 studies assessed the ORR in cancer patients with MA/MPE treated with H101, and there was heterogeneity among them (*I* ^2^ = 67.918%, *p* < 0.001). The pooled analysis illustrated an ORR of 69.9% (95%CI = 63.5%–76.4%) in these patients (**Figure 2**).

### 3.4 Subgroup analysis for ORR

**Figure 3:**
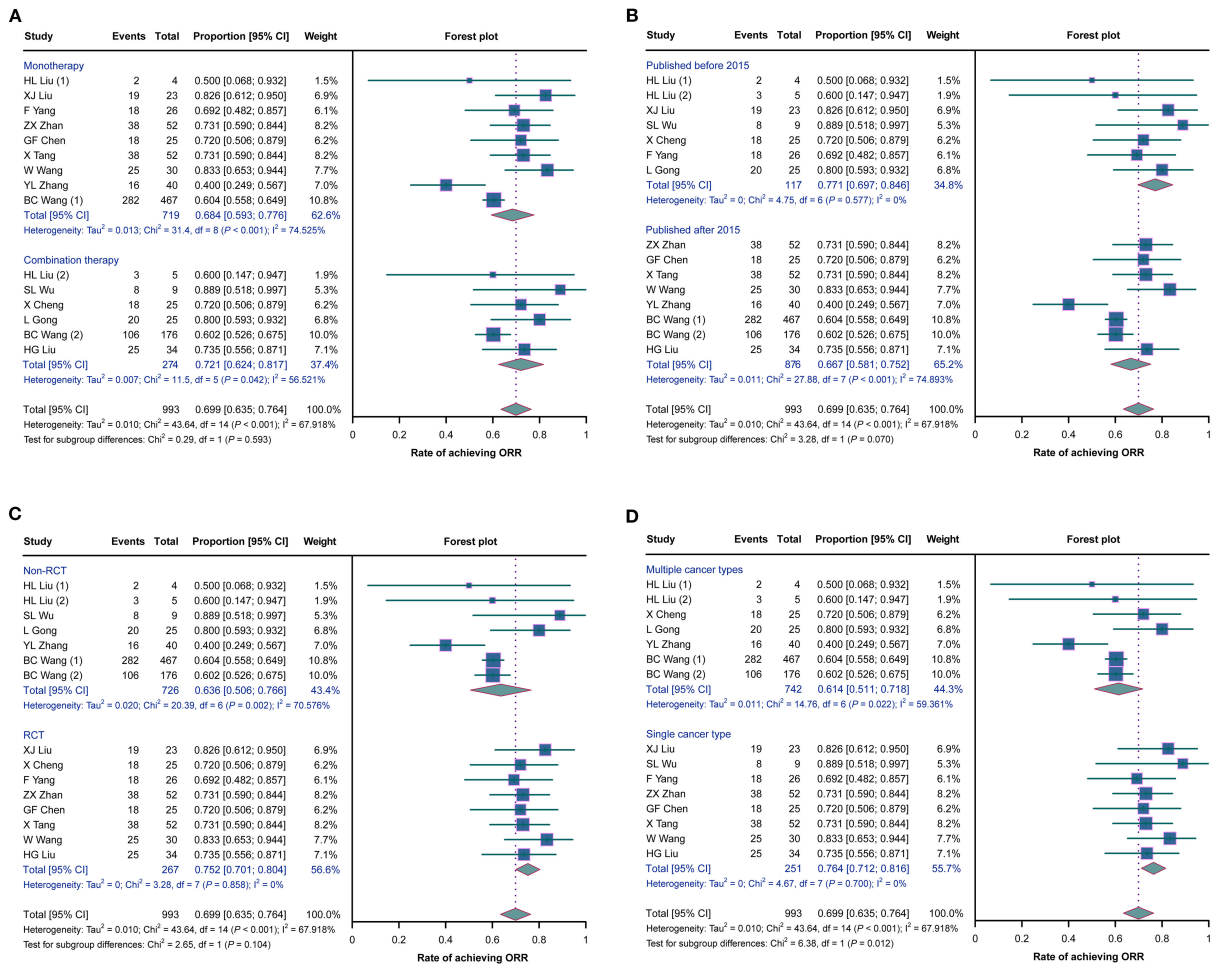
Forest plots of the subgroup analysis of the overall remission rates (ORRs) in cancer patients with malignant ascites (MA) or pleural effusion (MPE) treated with H101. **(A–D)** Comparisons of the ORRs between studies with monotherapy and those with combination therapy **(A)**, between studies published before 2015 and those published after 2015 **(B)**, between randomized controlled trials (RCTs) and non-RCTs **(C)**, and between studies with a single cancer type and those with multiple cancer types **(D)**.

No statistically significant differences in the ORRs were observed between studies with monotherapy and those with combination therapy (*p* = 0.593), between studies published before 2015 and those published after 2015 (*p* = 0.070), or between RCTs and non-RCTs (*p* = 0.104) (**Figures 3A–C**). However, the ORR was higher in studies with a single cancer type than in those with multiple cancer types (*p* = 0.012) (**Figure 3D**).

### 3.5 Adverse reactions

**Figure 4:**
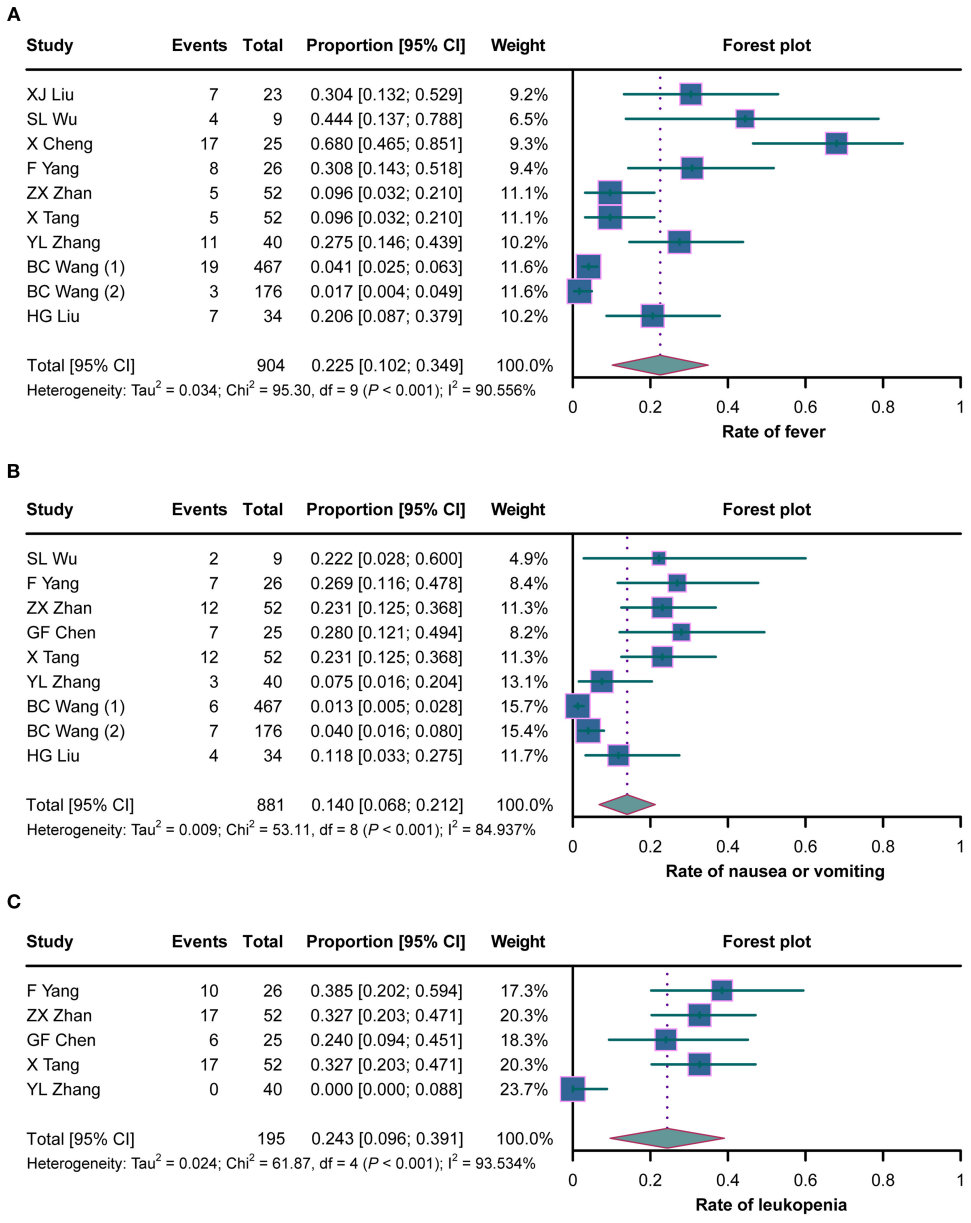
Forest plots of the adverse reactions in cancer patients with malignant ascites (MA) or pleural effusion (MPE) treated with H101. **(A–C)** Rates of fever **(A)**, nausea or vomiting **(B)**, and leukopenia **(C)** in cancer patients with MA/MPE treated with H101.

A total of nine studies evaluated the rate of fever in cancer patients with MA/MPE treated with H101, where heterogeneity existed (*I* ^2^ = 90.556%, *p* < 0.001). The pooled analysis showed that the rate of fever was 22.5% (95%CI = 10.2%–34.9%) (**Figure 4A**). There was heterogeneity among the eight studies that assessed the rate of nausea or vomiting (*I* ^2^ = 84.937%, *p* < 0.001). The pooled rate of nausea or vomiting was 14.0% (95%CI = 6.8%–21.2%) (**Figure 4B**). Five studies analyzed the rate of leukopenia, and there was heterogeneity among these studies (*I* ^2^ = 93.534%, *p* < 0.001). The pooled analysis disclosed that the rate of leukopenia was 24.3% (95%CI = 9.6%–39.1%) (**Figure 4C**).

The results of the subgroup analysis for adverse reactions in cancer patients with MA/MPE treated with H101 revealed that the rate of fever was higher in studies published before 2015 than in those published after 2015 (*p* = 0.001). The rate of nausea or vomiting was higher in RCTs than in non-RCTs and was also higher in studies with a single cancer type than in those with multiple cancer types (both *p* < 0.001). The rate of leukopenia was higher in RCTs than in non-RCTs and was also higher in studies with a single cancer type than in those with multiple cancer types (both *p* < 0.001) (**Supplementary Table S1**).

### 3.6 Meta-regression

Multivariate meta-regression analysis was conducted to further explore the sources of heterogeneity, including therapy type, publication year, study design, and cancer type.

For ORR, the model was statistically significant (*p* = 0.010), accounting for 70.51% of the heterogeneity, with a residual heterogeneity of 26.20%. However, no individual covariate reached significance.

For the rate of fever, publication year (after 2015 *vs*. before 2015) was related to its decreased rate (*β* = −1.863, *p* = 0.025), but the model was not statistically significant (*p* = 0.111), which accounted for 31.36% of the heterogeneity, with a residual heterogeneity of 87.75%.

For the rate of nausea or vomiting, there was no individual covariate affecting the result, although the model was statistically significant (*p* = 0.001). The model explained 77.01% of the heterogeneity, with a residual heterogeneity of 63.19%.

For the rate of leukopenia, the study design (RCT vs. non-RCT) was related to its increased rate (*β* = 3.601, *p* = 0.012). The model was statistically significant (*p* = 0.030), accounting for 100.00% of the heterogeneity, with a residual heterogeneity of 0.00% (**Supplementary Table S2**).

### 3.7 Publication bias and sensitivity analyses

Funnel plots and Begg’s test were used to assess publication bias. The funnel plots for the results of the ORR and of the rates of fever, nausea or vomiting, and leukopenia are shown in **Supplementary Figures S2A–D**. Begg’s test showed a publication bias in the rate of fever (*p* = 0.019), while there was no publication bias in the ORR, the rate of nausea or vomiting, or the rate of leukopenia (all *p* > 0.05) (**Table 2**). The publication bias in the rate of fever was corrected using the trim-and-fill method, and the adjusted rate of fever was 5.4% (95%CI = 0.0%–22.0%). After correction, no publication bias was observed in the rate of fever according to the Begg’s test (*p* = 0.803).

**Table 2:** Assessment of publication bias using Begg’s test.

Item	*p*-value	Bias estimate
ORR	0.804	−5.000
Fever	0.019	26.000
Nausea or vomiting	0.073	17.000
Leukopenia	0.795	1.000

*ORR*, overall remission rate.

The results of the sensitivity analyses indicated that the ORR, as well as the rates of fever, nausea or vomiting, and leukopenia, will not be influenced by omitting any study, indicating the high robustness of the results (**Table 3**).

**Table 3:** Sensitivity analysis via omitting studies one by one.

Omitted study	Proportion (95%CI)
ORR	
HL Liu (1)	0.702 (0.637–0.768)
HL Liu (2)	0.701 (0.635–0.767)
XJ Liu	0.690 (0.623–0.756)
SL Wu	0.688 (0.624–0.753)
X Cheng	0.698 (0.629–0.768)
F Yang	0.700 (0.631–0.769)
L Gong	0.692 (0.624–0.760)
ZX Zhan	0.697 (0.627–0.767)
GF Chen	0.698 (0.629–0.768)
X Tang	0.697 (0.627–0.767)
W Wang	0.688 (0.622–0.753)
YL Zhang	0.717 (0.661–0.772)
BC Wang (1)	0.711 (0.642–0.779)
BC Wang (2)	0.710 (0.642–0.779)
HG Liu	0.697 (0.627–0.767)
Fever	
XJ Liu	0.219 (0.083–0.355)
SL Wu	0.210 (0.083–0.338)
X Cheng	0.160 (0.076–0.243)
F Yang	0.219 (0.083–0.355)
ZX Zhan	0.243 (0.107–0.380)
X Tang	0.243 (0.107–0.380)
YL Zhang	0.222 (0.084–0.360)
BC Wang (1)	0.250 (0.118–0.382)
BC Wang (2)	0.252 (0.123–0.381)
HG Liu	0.230 (0.091–0.369)
Nausea or vomiting	
SL Wu	0.137 (0.061–0.212)
F Yang	0.127 (0.054–0.200)
ZX Zhan	0.128 (0.052–0.204)
GF Chen	0.126 (0.054–0.199)
X Tang	0.128 (0.052–0.204)
YL Zhang	0.153 (0.071–0.235)
BC Wang (1)	0.161 (0.088–0.234)
BC Wang (2)	0.159 (0.080–0.237)
HG Liu	0.146 (0.064–0.229)
Leukopenia	
F Yang	0.214 (0.049–0.379)
ZX Zhan	0.225 (0.046–0.403)
GF Chen	0.248 (0.064–0.431)
X Tang	0.225 (0.046–0.403)
YL Zhang	0.319 (0.246–0.392)

*CI*, confidence interval; *ORR*, overall remission rate

### 3.8 Risk of bias assessment

The Newcastle–Ottawa Scale was used to assess the risk of bias for five non-RCTs. The total score for these studies ranged from 7 to 9, suggesting that all of these studies were of high quality and that there was a low risk of bias (**Supplementary Table S3**).

The Cochrane ROB tool was used to assess the risk of bias for eight RCTs. The results showed that the risk of bias due to deviations from the intended interventions was unclear in the included studies. All of the studies were assessed as having “low risk” with regard to bias arising from the randomization process, bias due to missing outcome data and incomplete outcome data, and bias in the measurement of the outcome and the selection of the reported results (**Supplementary Table S4**).

A summary of the results of the risk of bias assessment is shown in **Supplementary Figure S3**.

## 4 Discussion

MA or MPE is a common complication that occurs in various cancers, resulting in poor prognosis for patients with cancer (29). Intraperitoneal/intrapleural perfusion therapy with chemotherapy drugs is the most common strategy for cancer patients with MA/MPE, but its efficacy is limited (27, 30, 31). Previous studies have reported ORRs of ascites or pleural effusion of 31.0%–50.0% in cancer patients with MA/MPE who received chemotherapy-based intraperitoneal/intrapleural perfusion therapy (27, 30, 31). This meta-analysis explored the efficacy of H101 in cancer patients with MA/MPE, which showed an ORR of 69.9%. The result of this meta-analysis was higher than that of the above studies (27, 30, 31), indicating fonc.2025.1592995that H101 may be a more effective treatment option than other intrapleural/intraperitoneal therapies for cancer patients with MA/MPE. A detailed explanation is as follows: 1) H101 could selectively replicate in tumor cells, inducing oncolytic responses to eliminate tumor cells on the surface of the thorax and peritoneum, thus reducing the formation of effusion (14, 25); or 2) H101 could regulate the tumor immune microenvironment to attract immune cells to kill tumor cells (24). Concurrently, a *post-hoc* power analysis was conducted to assess the sufficiency of the included sample size. Based on the pooled ORR of 0.699 (*n* = 993), with a null hypothesis proportion of 0.5 and a two-tailed *α* of 0.05, the estimated statistical power was 99.9%, indicating that this meta-analysis might have sufficient power to detect the observed effect size. However, more studies with large sample sizes are still required for further verification.

This meta-analysis also assessed the safety of H101 in cancer patients with MA/MPE, and the results revealed that the rates of fever, nausea or vomiting, and leukopenia were 22.5%, 14.0%, and 24.3%, respectively. These adverse events were controllable, indicating a good safety profile of H101 in cancer patients with MA/MPE. Notably, a previous study also assessed the safety of H101 in patients with MA, which showed that H101 was well tolerated, reporting pyrexia, fatigue, nausea, and abdominal pain as the most frequent adverse events (32). This previous study also verified the results of our meta-analysis to some extent (32). However, this study was not included in our meta-analysis as it did not evaluate the efficacy of H101.

The subgroup analysis revealed that the ORR and the rates of nausea or vomiting and leukopenia were higher in studies with a single cancer type than in those with multiple cancer types. This might be due to the fact that the weights used in the studies of Zhang (24) and Wang (25) were large, which might have interfered with the results to a certain degree. At the same time, this subgroup analysis was exploratory and required further verification. The rate of fever was higher in studies published before 2015 than in those published after 2015. However, this subgroup analysis was also exploratory, and the results might have been due to some underlying confounding factors (such as variations in the patient screening criteria or the care standards over time) rather than the publication year itself. The rate of nausea or vomiting was higher in RCTs than in non-RCTs. However, it was observed that the result in RCTs (21.1%) was more similar to the overall pooled result (14.0%) than that in non-RCTs (3.1%). This could be explained by the fact that, compared with non-RCTs, the design of RCTs is more rigorous; thus, the results of RCTs might be more representative of the actual situation. Moreover, the rate of leukopenia was also higher in RCTs than in non-RCTs, which might have been due to the large difference in the number of studies between the two subgroups: only one non-RCT reported the rate of leukopenia, which was 0.0%; thus, it could not represent the overall situation.

OVs have shown broad application prospects owing to their remarkable efficacy in the treatment of cancers (33). Currently, three OVs—H101, talimogene laherparepvec (T-VEC), and Delytact (G47Δ)—have been approved for commercial use (33). Although these OVs have been shown to exert antitumor effects, only H101 is currently demonstrated to have favorable efficacy in the management of cancers with MA/MPE in clinical practice. This finding highlights the significance of H101 within the global OV landscape. Notably, compared with traditional treatment methods for cancer with MA/MPE, the complex conditions for the transportation, storage, and handling of H101 might increase costs and could be a limitation for its clinical application (34). However, there is currently no specific study exploring the cost-effectiveness of H101.

In this meta-analysis, there was no publication bias in the ORR, the rate of nausea or vomiting, or the rate of leukopenia, while there was publication bias in the rate of fever. This bias was eliminated using the trim-and-fill method. The quality assessment exhibited the high quality of the included studies, and the sensitivity analyses demonstrated the high robustness of the results. However, some limitations still exist: 1) The number of included studies in this meta-analysis is relatively small, and the sample size of each included study is limited. Although an estimated statistical power of 99.9% indicated that this meta-analysis might have sufficient power to detect the observed effect size, more available studies with large sample sizes are still required for further verification; 2) Due to the lack of patient-centered outcomes (such as quality of life, long-term survival, and functional status), this meta-analysis did not evaluate these outcomes in cancer patients with MA/MPE who received H101; 3) Some of the included studies are non-RCTs, which might cause potential bias in the results; 4) Most of the included studies are published in Chinese, and the narrow geographical focus of these included studies might affect the generalizability of the results; 5) The ORR was not standardized across the included studies, which might influence the credibility of our results to some extent; 6) Although this meta-analysis revealed the efficacy and safety of H101 in cancer patients with MA/MPE, further basic experiments or clinical studies are required to offer novel mechanistic insights or comparison with other therapies; 7) Future studies could consider comparing the cost-effectiveness of H101 against other therapies; and 8) In the quality assessment of the included RCTs, the risk of bias due to deviations from the intended interventions was unclear, which is due to these RCTs not reporting whether deviations from interventions existed between the experimental group and the control group. This might potentially affect the results of this meta-analysis; for instance, it might increase the uncertainty of the results.

In conclusion, this meta-analysis shows that H101 may have good efficacy and favorable safety in the treatment of MA/MPE in patients with cancer, supporting its application in clinical management. However, most of the studies included in this meta-analysis are from China, and the generalizability of the results still requires further verification.
