# Supplementary material for: Recombinant human adenovirus type 5 administration for the treatment of malignant ascites or pleural effusion in cancer patients: a meta-analysis
Source: Front Oncol. 2025 Sep 17;15:1592995. doi: 10.3389/fonc.2025.1592995 (PMC12483890; doi:10.3389/fonc.2025.1592995)
Supplement: Supplementary file 1 [file DataSheet1.docx]

***Supplementary Materials***

**
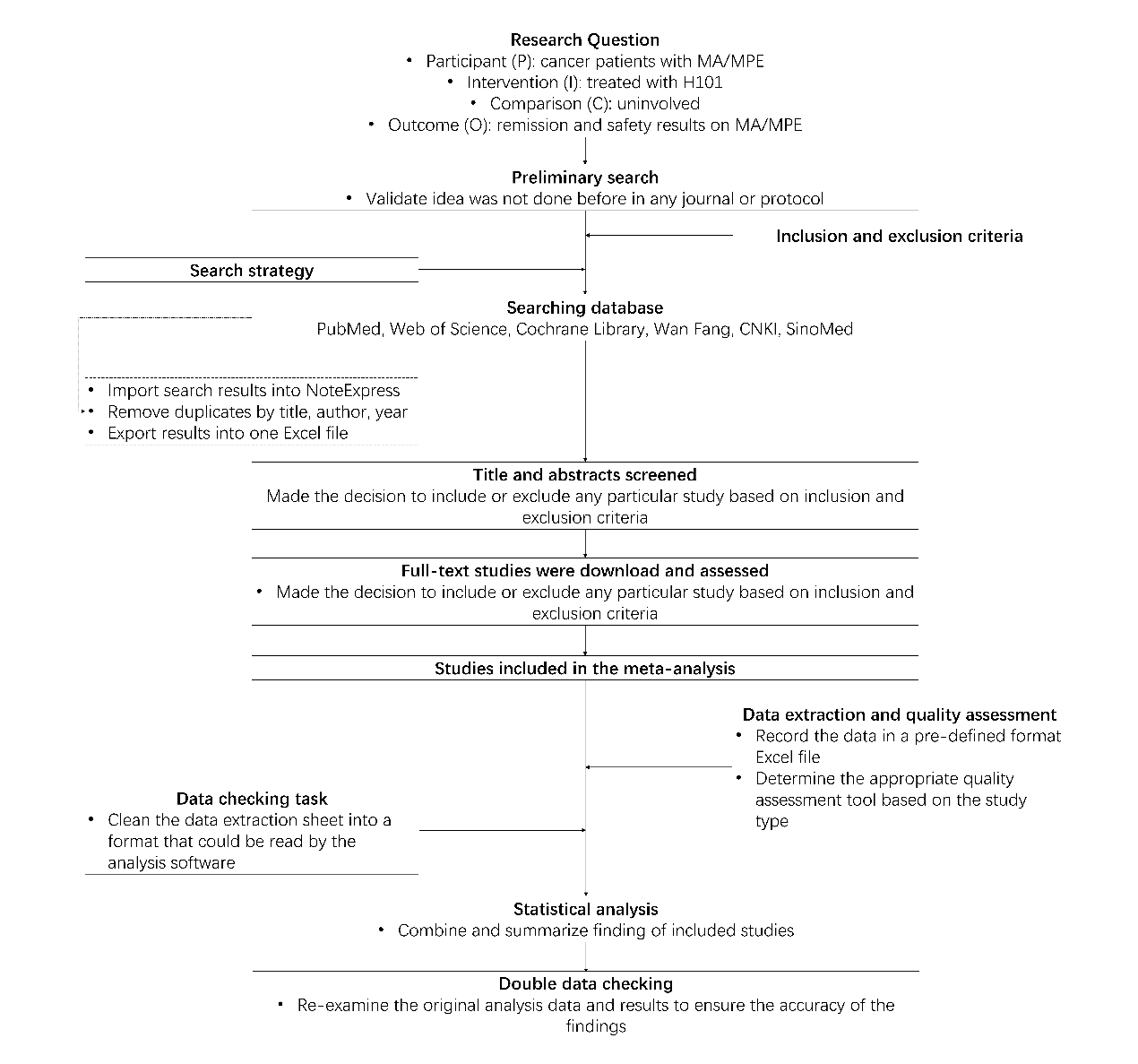
**

**Supplementary Figure 1.** Comprehensive methodological workflow flow.

**
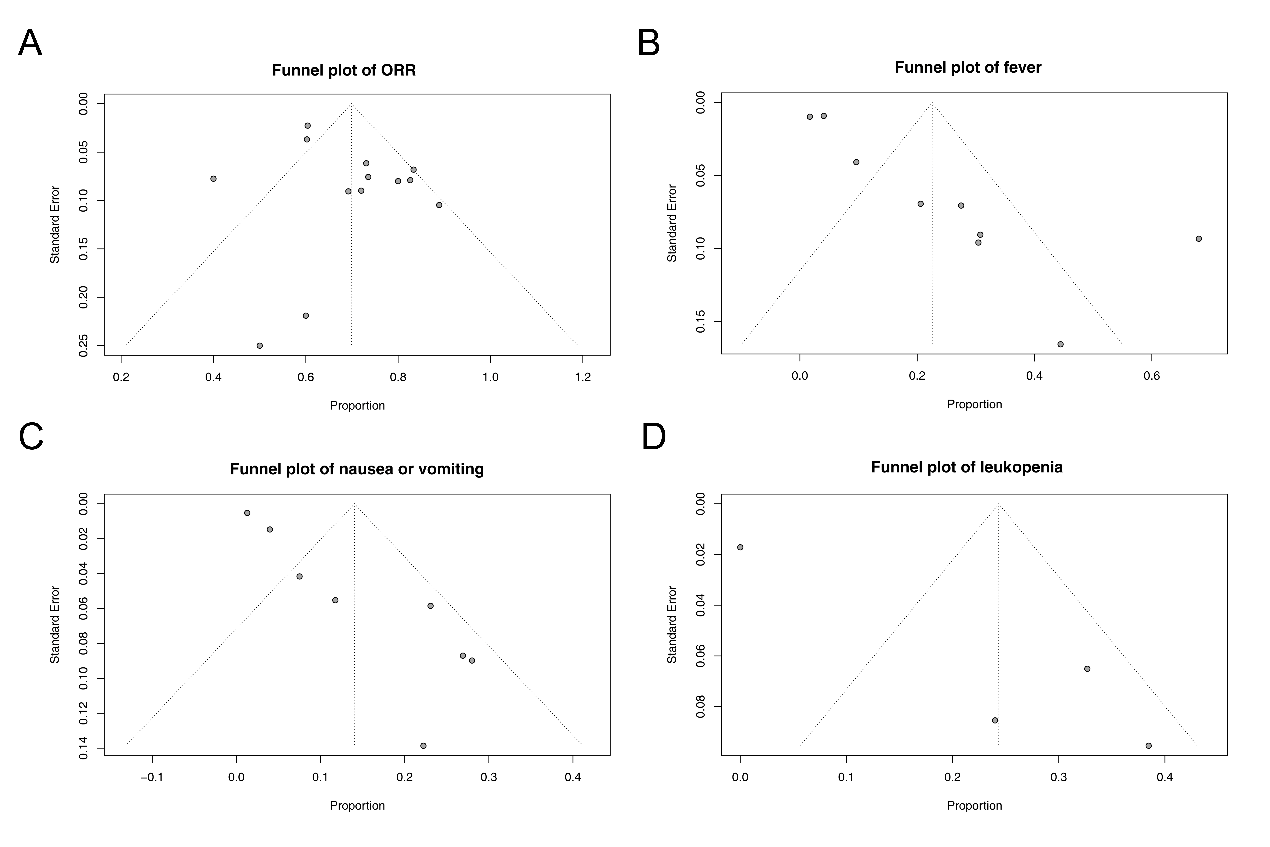
**

**Supplementary Figure 2.** Assessment of publication bias by funnel plots. The funnel plots for the results of ORR (**A**), the rate of fever (**B**), the rate of nausea or vomiting (**C**), and the rate of leukopenia (**D**).

**
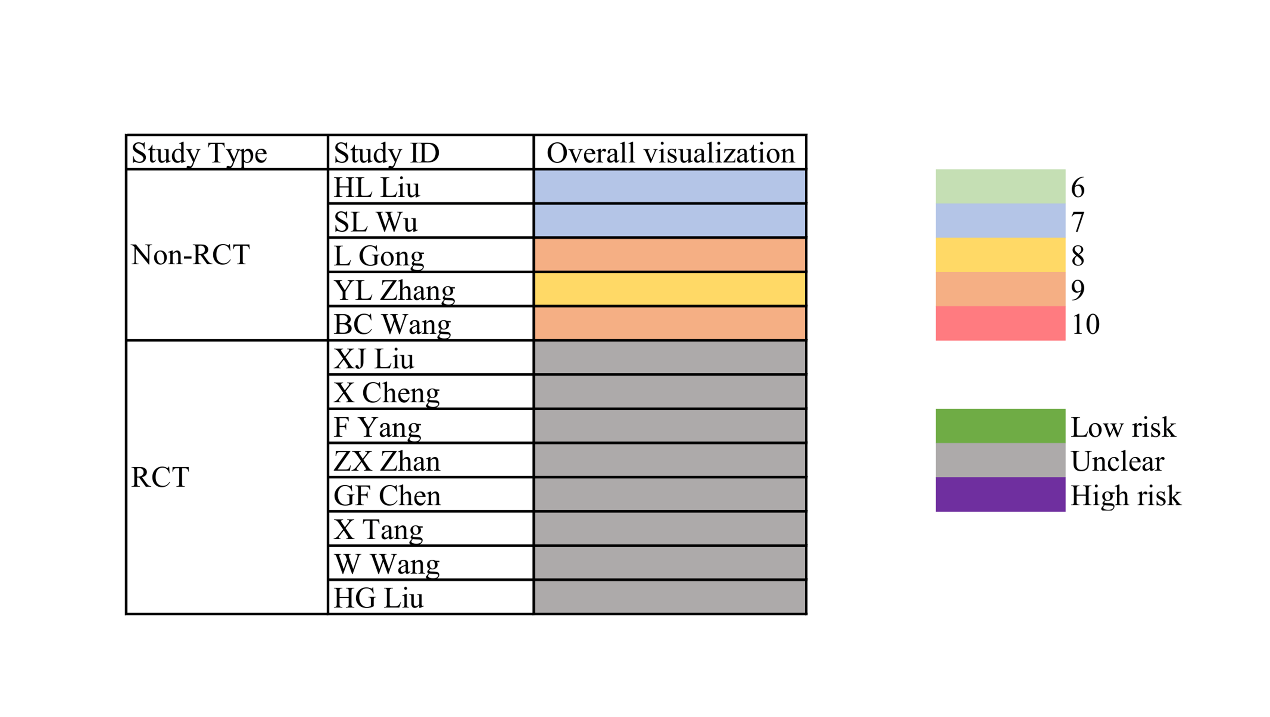
**

**Supplementary Figure 3.** Assessment of the risk of bias.

**Supplementary Table 1.** Subgroup analysis on fever, nausea or vomiting, and leukopenia.

| Subgroups | Fever | | | | Nausea or vomiting | | | | Leukopenia | | | |
| --- | --- | --- | --- | --- | --- | --- | --- | --- | --- | --- | --- | --- |
|  | No. of studies | | Total [95% CI] | *P* value | No. of studies | | Total [95% CI] | *P* value | No. of studies | | Total [95% CI] | *P* value |
| **Therapy type** |  |  | | 0.311 |  |  | | 0.159 |  |  | | (-) |
| Monotherapy | 6 | 0.162  [0.067; 0.258] | |  | 6 | 0.165  [0.068; 0.262] | |  | 5 | 0.243  [0.096; 0.391] | |  |
| Combination therapy | 4 | 0.322  [0.028; 0.617] | |  | 3 | 0.076  [0.000; 0.153] | |  | 0 | (-) | |  |
| **Published year** |  |  | | 0.001 |  |  | | 0.109 |  |  | | 0.181 |
| Before 2015 | 4 | 0.433  [0.243; 0.624] | |  | 2 | 0.256  [0.112; 0.400] | |  | 1 | 0.385  [0.198; 0.572] | |  |
| After 2015 | 6 | 0.102  [0.031; 0.172] | |  | 7 | 0.122  [0.046; 0.199] | |  | 4 | 0.214  [0.049; 0.379] | |  |
| **Study type** |  |  | | 0.326 |  |  | | <0.001 |  |  | | <0.001 |
| Non-RCT | 6 | 0.271  [0.101; 0.441] | |  | 4 | 0.031  [0.002; 0.060] | |  | 1 | 0.000  [0.000; 0.034] | |  |
| RCT | 4 | 0.151  [0.000; 0.320] | |  | 5 | 0.211  [0.148; 0.273] | |  | 4 | 0.319  [0.246; 0.392] | |  |
| **Cancer type** |  |  | | 0.770 |  |  | | <0.001 |  |  | | <0.001 |
| Multiple | 4 | 0.243  [0.000; 0.537] | |  | 3 | 0.028  [0.001; 0.055] | |  | 1 | 0.000  [0.000; 0.034] | |  |
| Single | 6 | 0.197  [0.103; 0.291] | |  | 6 | 0.211  [0.151; 0.270] | |  | 4 | 0.319  [0.246; 0.392] | |  |

CI, confidence interval; RCT, randomized controlled trial.

**Supplementary Table 2**. Multivariate meta-regression analysis for ORR, fever, nausea or vomiting, and leukopenia.

| Items | β | 95% CI | | *P* value (Factor) |
| --- | --- | --- | --- | --- |
|  |  | Lower | Upper |  |
| **ORR** |  |  |  |  |
| Therapy type (Combination therapy vs. Monotherapy) | 0.282 | -0.217 | 0.782 | 0.268 |
| Publication year (After 2015 vs. Before 2015) | -0.354 | -0.926 | 0.217 | 0.224 |
| Study design (RCT vs. Non-RCT) | 0.005 | -0.976 | 0.986 | 0.992 |
| Cancer type (Single vs. Multiple) | 0.767 | -0.230 | 1.765 | 0.132 |
| I^2^ | 26.20% | | | |
| R^2^ | 70.51% | | | |
| *P* value (Model) | 0.010 | | | |
| **Fever** |  |  |  |  |
| Therapy type (Combination therapy vs. Monotherapy) | 0.263 | -1.319 | 1.845 | 0.745 |
| Publication year (After 2015 vs. Before 2015) | -1.863 | -3.494 | -0.231 | 0.025 |
| Study design (RCT vs. Non-RCT) | 0.735 | -1.215 | 2.685 | 0.460 |
| Cancer type (Single vs. Multiple) | -0.335 | -2.261 | 1.592 | 0.734 |
| I^2^ | 87.75% | | | |
| R^2^ | 31.36% | | | |
| *P* value (Model) | 0.111 | | | |
| **Nausea or vomiting** |  |  |  |  |
| Therapy type (Combination therapy vs. Monotherapy) | -0.239 | -1.404 | 0.927 | 0.688 |
| Publication year (After 2015 vs. Before 2015) | -0.258 | -1.788 | 1.272 | 0.741 |
| Study design (RCT vs. Non-RCT) | 0.016 | -2.590 | 2.621 | 0.991 |
| Cancer type (Single vs. Multiple) | 2.070 | -0.652 | 4.791 | 0.136 |
| I^2^ | 63.19% | | | |
| R^2^ | 77.01% | | | |
| *P* value (Model) | 0.001 | | | |
| **Leukopenia** |  |  |  |  |
| Therapy type (Combination therapy vs. Monotherapy) | (-) | (-) | (-) | (-) |
| Publication year (After 2015 vs. Before 2015) | -0.324 | -1.198 | 0.551 | 0.468 |
| Study design (RCT vs. Non-RCT) | 3.601 | 0.787 | 6.415 | 0.012 |
| Cancer type (Single vs. Multiple) | (-) | (-) | (-) | (-) |
| I^2^ | 0.00% | | | |
| R^2^ | 100.00% | | | |
| *P* value (Model) | 0.030 | | | |

CI, confidence interval; ORR, objective response rate; RCT, randomized controlled trial.

**Supplementary Table 3.** Risk of bias for non-RCT study via Newcastle-Ottawa Scale tool.

| Study ID | Newcastle-Ottawa Scale tool (non-RCT study) | | | |
| --- | --- | --- | --- | --- |
|  | Selection | Comparison | Outcome | Total |
| HL Liu | 4 | 1 | 2 | 7 |
| SL Wu | 4 | 1 | 2 | 7 |
| L Gong | 4 | 2 | 3 | 9 |
| YL Zhang | 4 | 1 | 3 | 8 |
| BC Wang | 4 | 2 | 3 | 9 |

RCT, randomized controlled trial.

**Supplementary Table 4.** Risk of bias for RCT study via Cochrane ROB tool.

| Study ID | Cochrane ROB tool (RCT study) | | | | | |
| --- | --- | --- | --- | --- | --- | --- |
|  | Bias arising from the randomization process | Bias due to deviations from intended interventions | Bias due to missing outcome data | Bias in measurement of the outcome | Incomplete outcome data | Bias in selection of the reported result |
| XJ Liu | Low risk | Unclear | Low risk | Low risk | Low risk | Low risk |
| X Cheng | Low risk | Unclear | Low risk | Low risk | Low risk | Low risk |
| F Yang | Low risk | Unclear | Low risk | Low risk | Low risk | Low risk |
| ZX Zhan | Low risk | Unclear | Low risk | Low risk | Low risk | Low risk |
| GF Chen | Low risk | Unclear | Low risk | Low risk | Low risk | Low risk |
| X Tang | Low risk | Unclear | Low risk | Low risk | Low risk | Low risk |
| W Wang | Low risk | Unclear | Low risk | Low risk | Low risk | Low risk |
| HG Liu | Low risk | Unclear | Low risk | Low risk | Low risk | Low risk |

RCT, randomized controlled trial.
