# Supplementary material for: Recombinant human adenovirus type 5 administration for the treatment of malignant ascites or pleural effusion in cancer patients: a meta-analysis
Source: Front Oncol. 2025 Sep 17;15:1592995. doi: 10.3389/fonc.2025.1592995 (PMC12483890; doi:10.3389/fonc.2025.1592995)
Supplement: Supplementary file 2 [file DataSheet2.docx]

***Appendix 1.*** Full search strategy in PubMed.

The detailed PubMed search strategy used for the mete-analysis was presented below. The search was conducted up to October 1, 2024.

Full query: ((cancer) OR (carcinoma) OR (neoplasms)) AND ((malignant ascites) OR (pleural effusion)) AND ((human type 5 recombinant adenovirus) OR (oncolytic viruses) OR (H101))

Details: ("cancer s"[All Fields] OR "cancerated"[All Fields] OR "canceration"[All Fields] OR "cancerization"[All Fields] OR "cancerized"[All Fields] OR "cancerous"[All Fields] OR "neoplasms"[MeSH Terms] OR "neoplasms"[All Fields] OR "cancer"[All Fields] OR "cancers"[All Fields] OR ("carcinoma"[MeSH Terms] OR "carcinoma"[All Fields] OR "carcinomas"[All Fields] OR "carcinoma s"[All Fields]) OR ("neoplasm s"[All Fields] OR "neoplasms"[MeSH Terms] OR "neoplasms"[All Fields] OR "neoplasm"[All Fields])) AND ((("malign"[All Fields] OR "malignance"[All Fields] OR "malignances"[All Fields] OR "malignant"[All Fields] OR "malignants"[All Fields] OR "malignities"[All Fields] OR "malignity"[All Fields] OR "malignization"[All Fields] OR "malignized"[All Fields] OR "maligns"[All Fields] OR "neoplasms"[MeSH Terms] OR "neoplasms"[All Fields] OR "malignancies"[All Fields] OR "malignancy"[All Fields]) AND ("ascite"[All Fields] OR "ascites"[MeSH Terms] OR "ascites"[All Fields] OR "ascitic"[All Fields])) OR ("pleural effusion"[MeSH Terms] OR ("pleural"[All Fields] AND "effusion"[All Fields]) OR "pleural effusion"[All Fields])) AND ((("human s"[All Fields] OR "humans"[MeSH Terms] OR "humans"[All Fields] OR "human"[All Fields]) AND "type"[All Fields] AND "5"[All Fields] AND ("f8 protein human"[Supplementary Concept] OR "f8 protein human"[All Fields] OR "recombinate"[All Fields] OR "recombinant"[All Fields] OR "recombinants"[All Fields] OR "recombinated"[All Fields] OR "recombinates"[All Fields] OR "recombination, genetic"[MeSH Terms] OR ("recombination"[All Fields] AND "genetic"[All Fields]) OR "genetic recombination"[All Fields] OR "recombination"[All Fields] OR "recombinations"[All Fields] OR "recombinational"[All Fields] OR "recombinative"[All Fields] OR "recombine"[All Fields] OR "recombined"[All Fields] OR "recombineered"[All Fields] OR "recombineering"[All Fields] OR "recombines"[All Fields] OR "recombining"[All Fields]) AND ("adenoviridae"[MeSH Terms] OR "adenoviridae"[All Fields] OR "adenovirus"[All Fields] OR "adenoviridae infections"[MeSH Terms] OR ("adenoviridae"[All Fields] AND "infections"[All Fields]) OR "adenoviridae infections"[All Fields])) OR ("oncolytic viruses"[MeSH Terms] OR ("oncolytic"[All Fields] AND "viruses"[All Fields]) OR "oncolytic viruses"[All Fields]) OR "H101"[All Fields])
